# Supplementary figures and images for: Phage S144, a New Polyvalent Phage Infecting Salmonella spp. and Cronobacter sakazakii
Source: Int J Mol Sci. 2020 Jul 22;21(15):5196. doi: 10.3390/ijms21155196 (PMC7432712; doi:10.3390/ijms21155196)

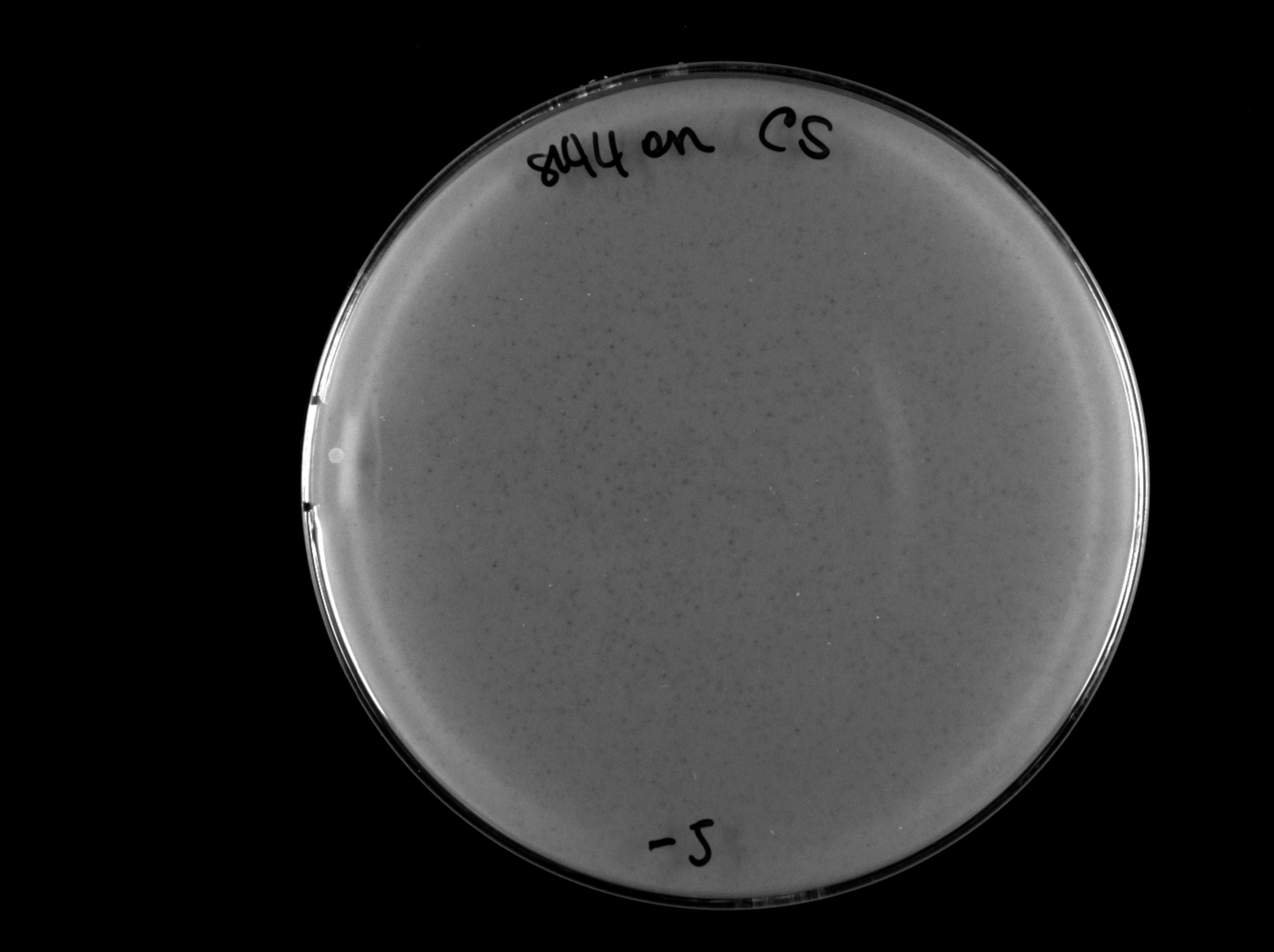

Supplement: Supplementary file 1 [file ijms-21-05196-s001.zip › supplementary material/S144 on CS.tif]

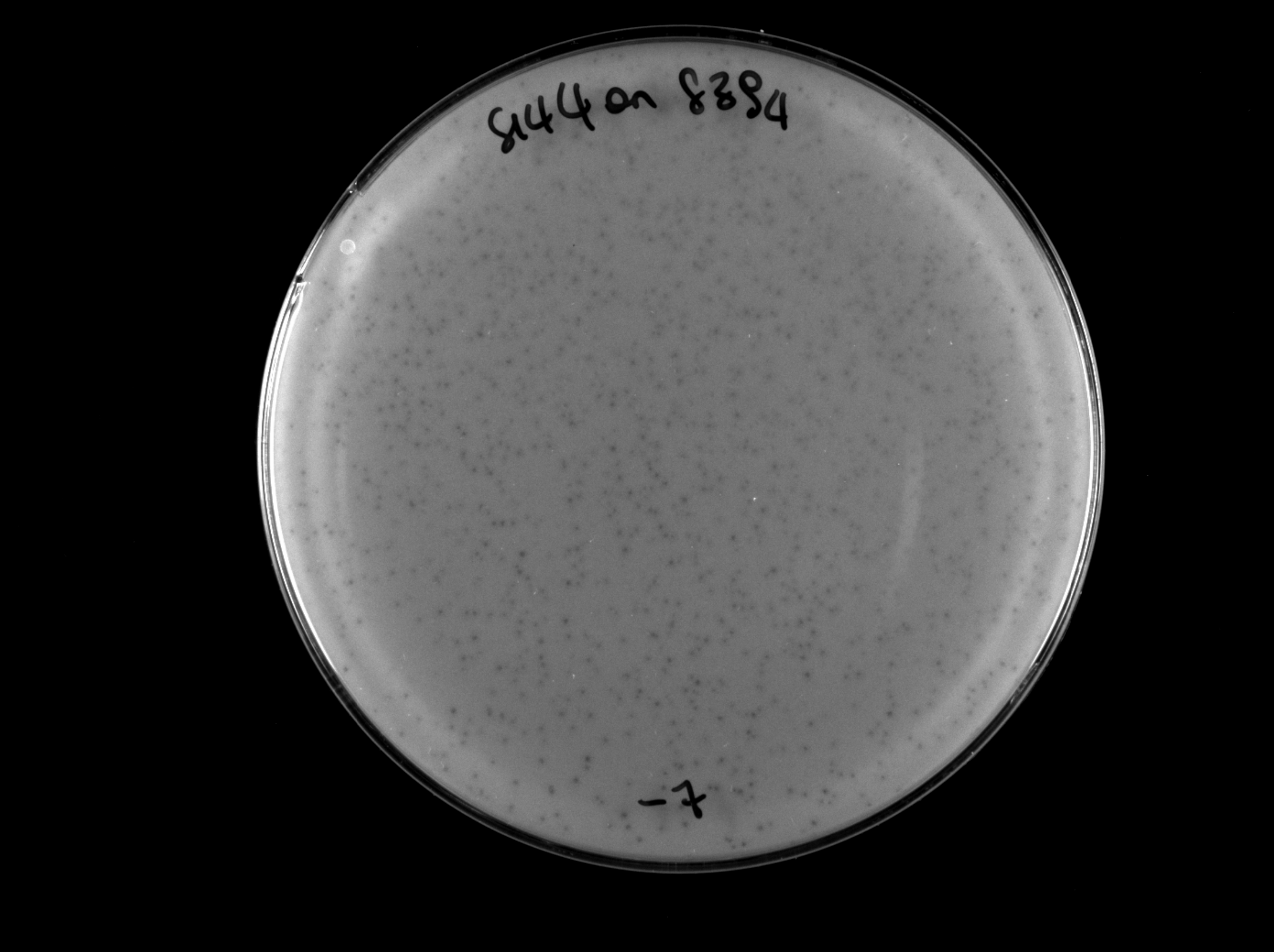

Supplement: Supplementary file 1 [file ijms-21-05196-s001.zip › supplementary material/S144 on S394.tif]

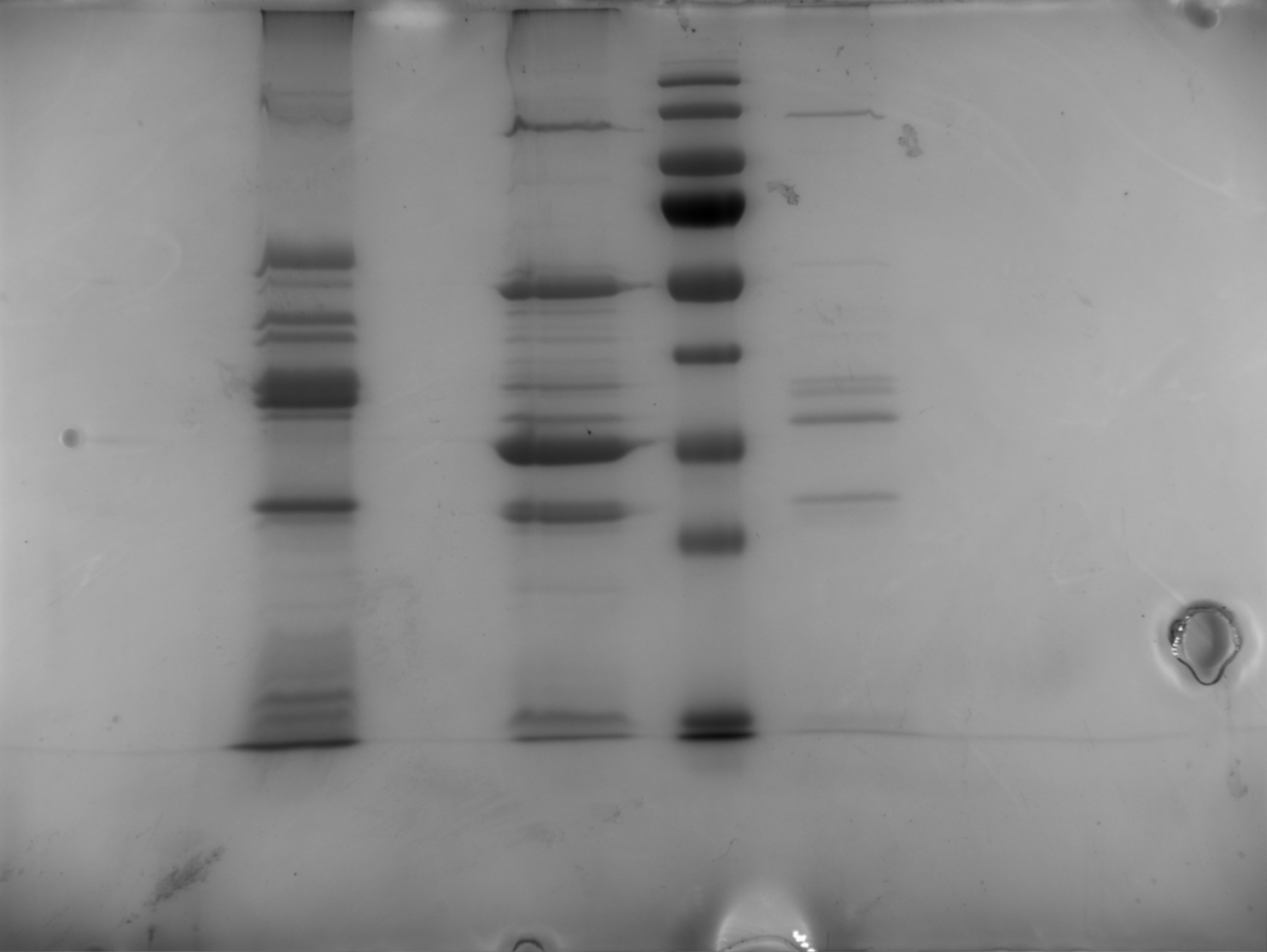

Supplement: Supplementary file 1 [file ijms-21-05196-s001.zip › supplementary material/Figure A5.jpg]

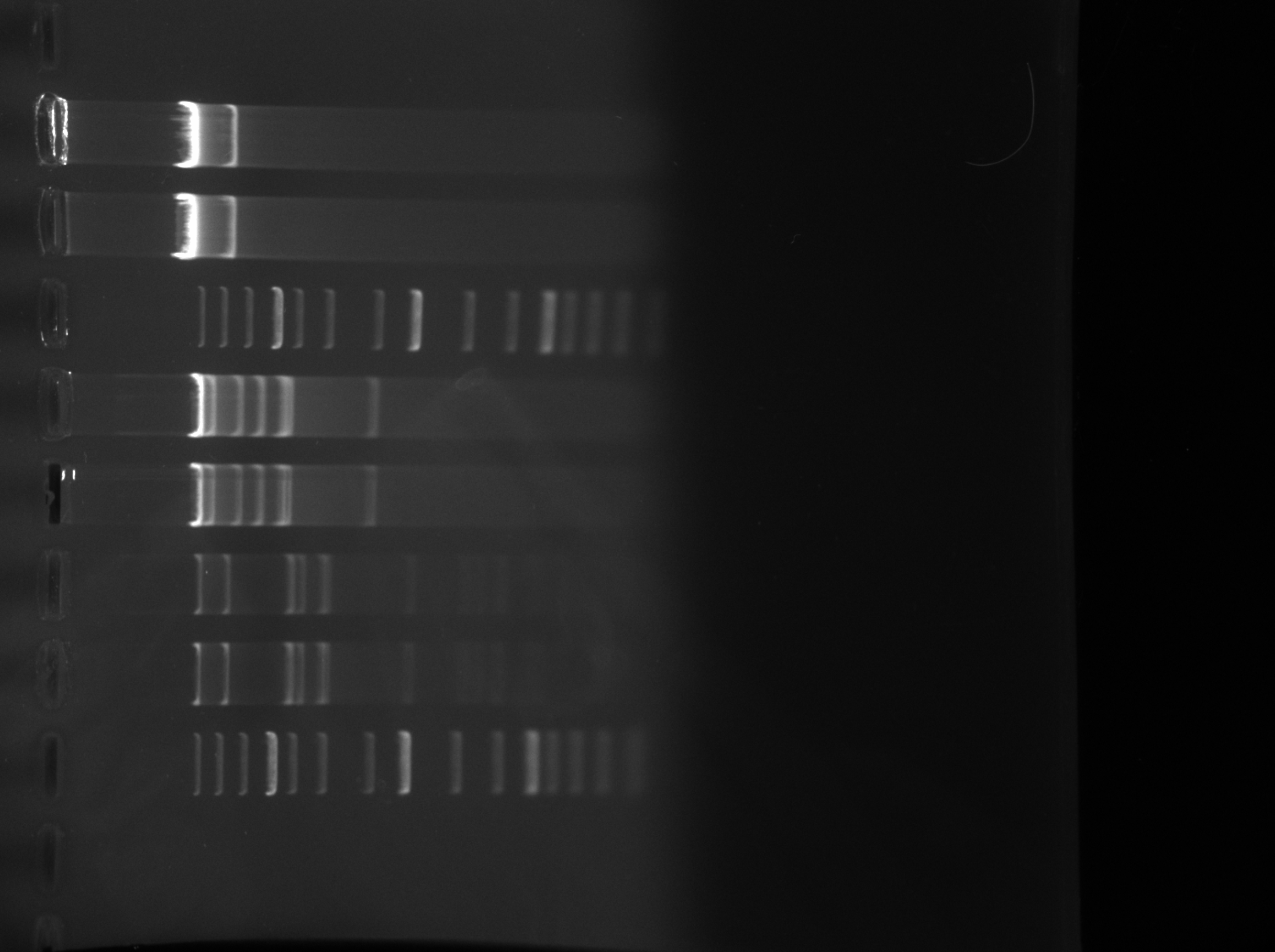

Supplement: Supplementary file 1 [file ijms-21-05196-s001.zip › supplementary material/Figure A4 RE SspI and PacI.tif]

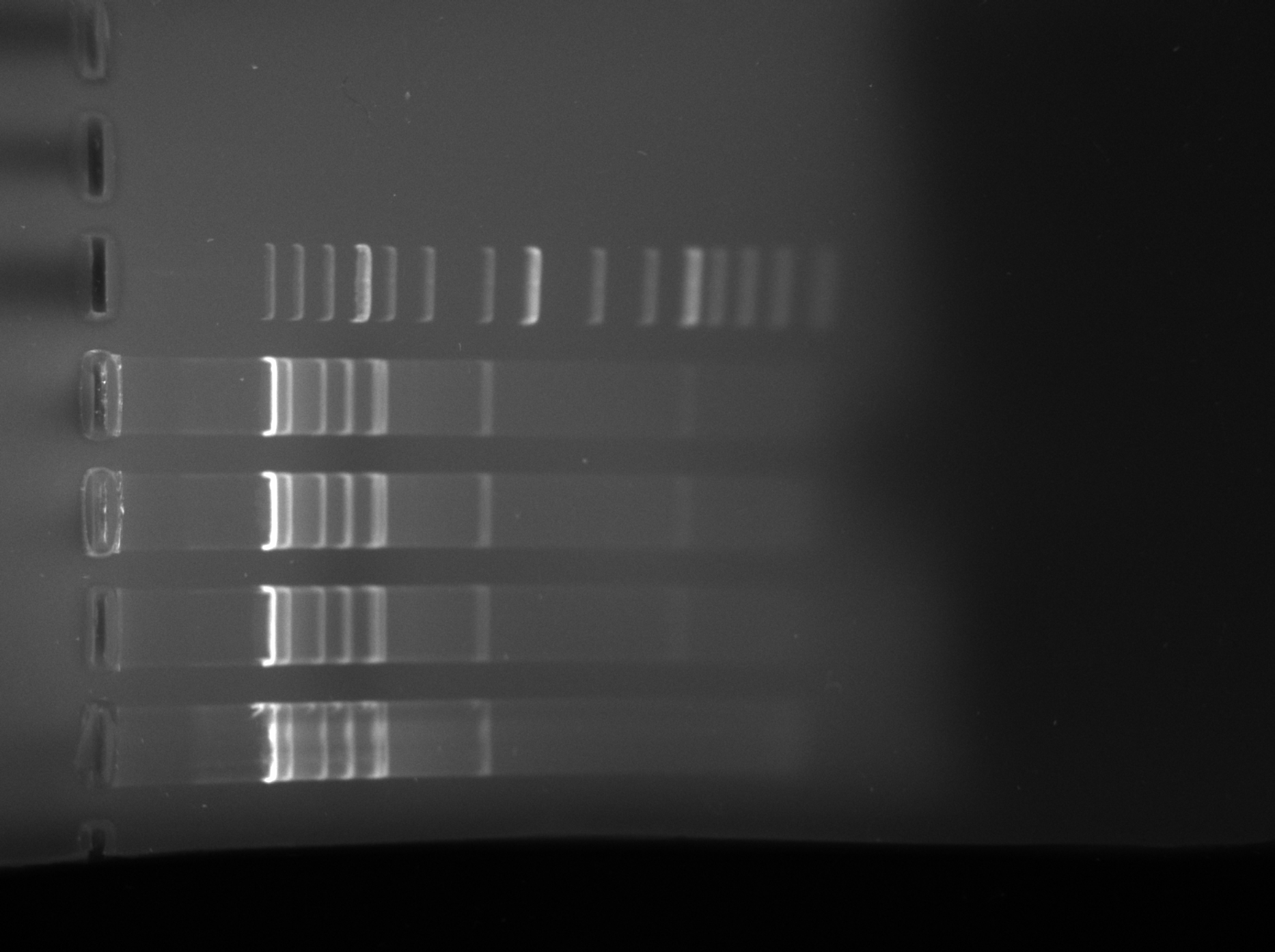

Supplement: Supplementary file 1 [file ijms-21-05196-s001.zip › supplementary material/Figure A4 RE SspI+PacI.tif]

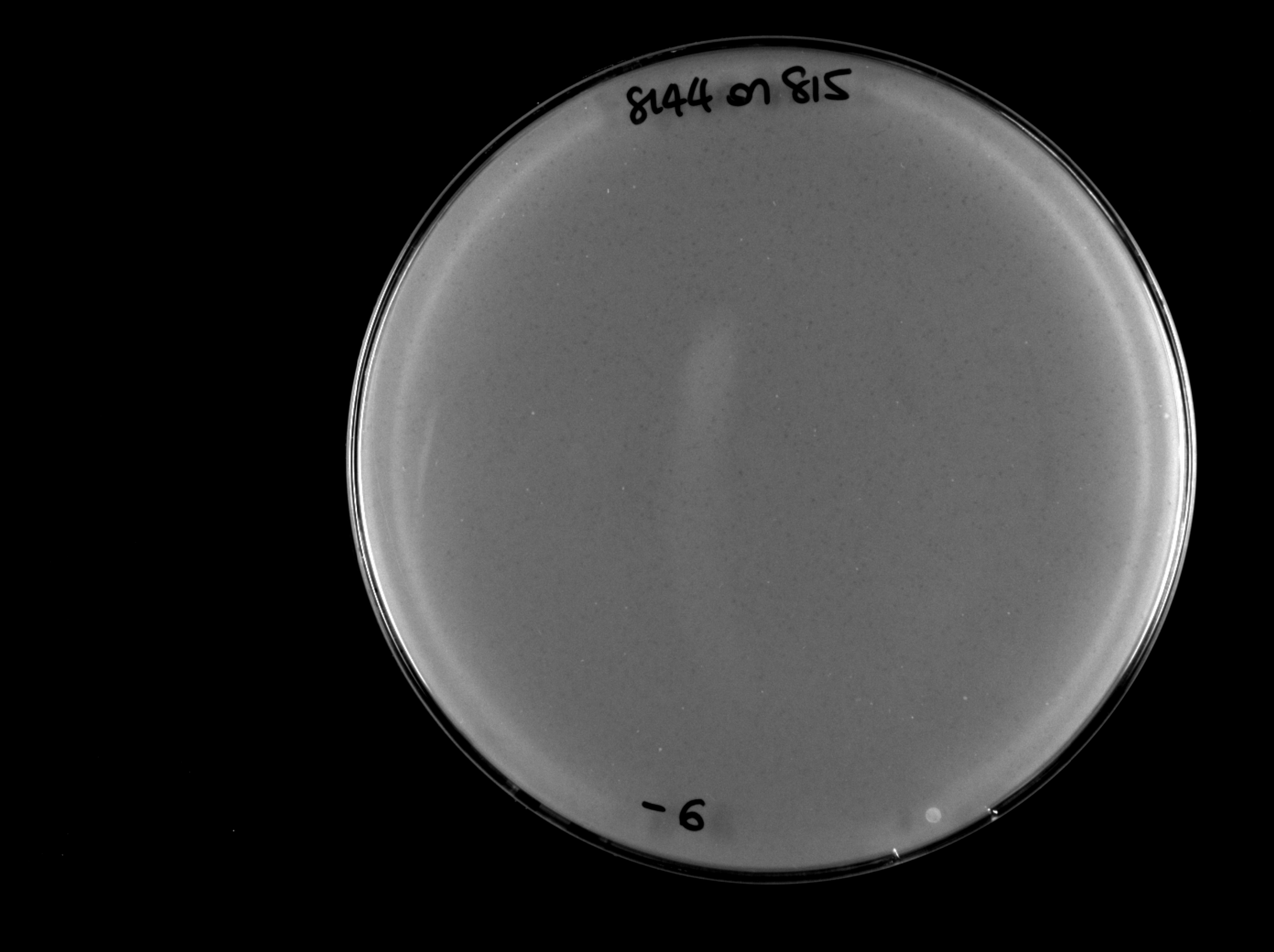

Supplement: Supplementary file 1 [file ijms-21-05196-s001.zip › supplementary material/S144 on S15.tif]
